# Improvement in Gait Variability over 24 Months in an Individual with Hemiparesis following Stroke: A Case Report

**DOI:** 10.1298/ptr.25-E10380

**Published:** 2026-02-06

**Authors:** Taishi KIKKAWA, Tsubasa MITSUTAKE, Takeshi IMURA, Yu INOUE, Ryo TANAKA

**Affiliations:** 1 Department of Rehabilitation, Ushioda General Hospital, Japan; 2 Graduate School of Humanities and Social Sciences, Hiroshima University, Japan; 3 Clinical Research Center, Saga University Hospital, Japan; 4 Department of Neurorehabilitation and Emotional Science, Graduate School of Biomedical and Health Sciences, Hiroshima University, Japan; 5 Major of Physical Therapy, Department of Human Science, School of Human Science, Kibi International University, Japan

**Keywords:** Stroke, Gait variability, Independent walking

## Abstract

**Objectives:**

Gait variability, related to gait stability, is crucial because it is associated with the risk of falls in patients with hemiparesis following stroke. However, there is a lack of understanding regarding changes in gait variability over time in patients with stroke. This case report aimed to describe the improvement in gait variability over 24 months in an individual with hemiparesis following stroke.

**Case Description:**

A 34-year-old man was admitted to the hospital with a diagnosis of cerebral infarction due to atherosclerosis. The patient presented with severe motor paralysis of the right extremities, scoring 6 and 13 on the Fugl–Meyer Assessment for the upper and lower extremities, respectively. Data collection for patient examination started at 1 month post-stroke. Two measures were employed for quantifying gait variability: the Gait Variability Index (GVI) and the coefficient of variation (CV). Nine spatiotemporal parameters were collected for calculating the GVI and CV.

**Results:**

The patient was discharged after 5 months post-stroke. At 24 months, he was able to walk independently outdoors without using a cane or an ankle–foot orthosis. Improvements in the GVI and CV were observed during the first 4 months following stroke; however, no marked changes were noted thereafter. At 24 months, the CV for step length, single support time, and swing time demonstrated the most marked improvements.

**Conclusions:**

Gait variability in this patient showed the most improvement during the subacute phase. Additionally, improvement in gait variability may be the basis for achieving independent walking, and future research is warranted.

## Introduction

Achieving independent walking is crucial for patients with hemiparesis following stroke. Many people with hemiparesis following stroke experience challenges with walking^1)^. The rehabilitation goal for these individuals encompasses regaining independent walking to move around in different locations^2)^. Considering the prevalence of gait instability in patients with stroke^3)^, restoring a stable gait is essential for achieving independent walking.

Gait variability is an indicator related to gait stability. Gait stability is defined as a gait that does not fall over despite perturbations, with the requirement of limiting the small perturbations that occur with each stride^4)^. Gait variability, defined as the stride-to-stride variation of spatiotemporal parameters during walking^5)^, is involved in gait stability to suppress and recover from perturbations during walking^4,6)^. Compared with healthy control limbs, patients with hemiparesis following stroke exhibit increased variability in stride time and step length for both paretic and nonparetic limbs^7–9)^. Such variability has been associated with a higher risk of falls^10,11)^, as well as lower levels of self-efficacy^12–14)^. Therefore, gait variability may reflect gait characteristics that are not entirely captured by walking speed alone.

Despite the importance of gait variability, there is a lack of understanding of the process by which it improves. In patients with stroke, a decrease in gait variability is considered an improvement. Critical questions persist, including the duration of gait variability changes in patients with stroke and which specific spatiotemporal gait parameters demonstrate improvement over time. Although immediate improvements in gait variability have been reported^15,16)^, evidence on long-term progression is lacking. In particular, it remains unclear whether gait variability follows a similar long-term course to gait characteristics such as walking speed. Furthermore, there is no consensus on which spatiotemporal gait parameters are most closely associated with achieving independent walking. Longitudinal studies on the improvement process in gait variability may offer valuable information for patients with stroke beyond this specific case. This case report aimed to describe the improvement of gait variability over 24 months in an individual with hemiparesis following stroke.

## Case Description

**Fig. 1.**
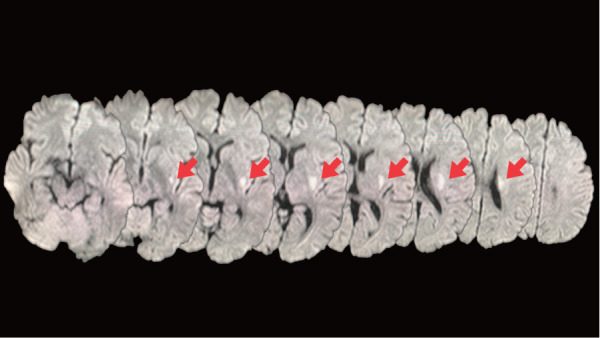
Diffusion-weighted image at onset. The arrows indicate the hyperintense lesion in the left lateral striatum.

This report was prepared following the CARE guidelines. The patient was a 34-year-old male who was admitted to the hospital with a diagnosis of cerebral infarction due to atherosclerosis. The patient was 170 cm tall, weighed 104 kg, and had a body mass index (BMI) of 36.1 kg/m^2^. The patient’s medical history included hypertension, dyslipidemia, and hyperhomocysteinemia. Before the stroke, the patient could independently perform daily activities and worked as an office employee. Magnetic resonance imaging on admission revealed a hyperintense lesion in the left lateral striatum (Fig. 1). The patient stated, “I cannot move my right limbs and feel as though they do not belong to me. Standing or walking feels frightening.” The patient was treated with antiplatelet, antihypertensive, lipid-lowering, and folic acid preparations.

### Physical therapy evaluation and intervention

At 3 days following stroke onset, the rehabilitation process started, and physical therapy interventions were initiated in the rehabilitation ward at 23 days post-stroke. The patient had severe right-sided hemiparesis, with scores of 6 and 13 for the upper and lower extremities on the Fugl–Meyer Assessment (FMA)^17)^. The sensory assessment revealed a slightly decreased light touch sensation on the paretic side. The Mini-Mental State Examination score was 30. The patient’s walking independence was classified as level 1 on the Functional Ambulation Category (FAC)^18)^. The 6-minute walk test (6MWT) was completed with moderate assistance, covering a total distance of 200 m using a cane and an ankle–foot orthosis (AFO). The Berg Balance Scale (BBS) score was 33, indicating a fall risk^19)^. The Japanese version of the Activities-specific Balance Confidence scale (ABC-J) indicated low balance confidence for indoor and outdoor activities, with a mean of 8.8%^20)^.

**Fig. 2.**
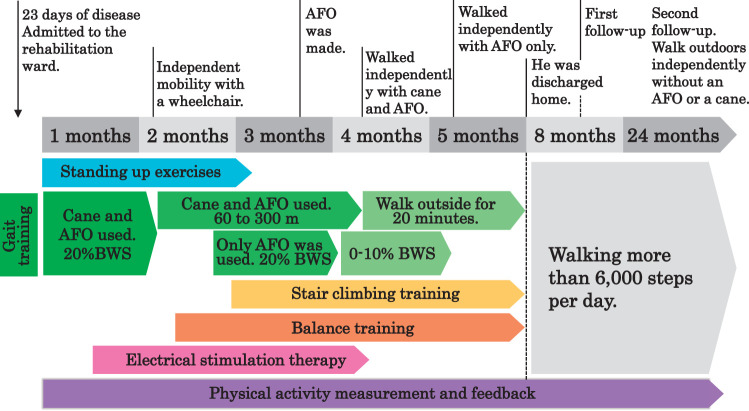
Physical therapy content and mobility timeline. AFO, ankle–foot orthosis; BWS, body weight support

Figure 2 depicts the physical therapy interventions administered in the hospital setting and the mobility course. The physical therapy program encompassed various exercises, including standing exercises, electrical stimulation for ankle dorsiflexion, gait training, balance training, and stair climbing. Gait training with body weight support (BWS) using a harness was implemented owing to the patient’s high risk of falling. One month post-stroke, the patient began gait training using a cane and at 20% BWS. Two months post-stroke, gait training using a cane and AFO without the harness and gait training without a cane at 20% BWS began. Four months post-stroke, outdoor walking using a cane and AFO and gait training without AFO at 0%–10% BWS were performed. At 1 month post-stroke, the patient began tracking his daily step count as a measure of physical activity (PA) using the Apple Health app (Apple, Cupertino, CA, USA) on his iPhone. PA was documented as the average daily step count over 5 weekdays, between 9 AM and 6 PM. Along with physical therapy, the patient underwent occupational therapy and speech–language–hearing therapy. These interventions were conducted daily for durations ranging from 40 to 180 min, 7 days a week, until 5 months post-stroke.

### Gait variability analysis

To analyze the variability of the spatiotemporal gait parameters, the Walk-Way MW-1000 electronic walkway (2.4 m long; ANIMA, Tokyo, Japan) was used, with a 100-Hz sampling frequency. The reliability of the Walk-Way MW-1000 has been reported with intraclass correlation coefficients of 0.923–0.945 for walking speed^21)^. This patient walked on the walkway at a self-selected comfortable speed, using a cane or an AFO as needed. During data collection, the patient walked 4–12 walking trials, with a total of 10 strides recorded. The timing of each initial contact and toe-off, as well as the toe and heel locations, was automatically extracted using the Walk-Way MW-1000 software.

Two methods were employed for quantifying gait variability. First, the Gait Variability Index (GVI) was calculated using an Excel spreadsheet, following the methodology of Gouelle and Megrot^22)^. The GVI is a composite index of variability that uses multiple spatiotemporal parameters during gait to provide a single score. Stride velocity, stride time, step time, swing time, stance time, single support time, double support time, stride length, and step length were the required parameters. A GVI score of 100 represents the average score in a healthy reference group and indicates low gait variability^22)^. For the patient, progress was assessed using the average GVI score of the paretic side, the nonparetic side, and both sides combined. Second, the variability in the nine spatiotemporal parameters was measured using the coefficient of variation (CV), calculated as (standard deviation/mean) × 100 (%). Furthermore, walking speed was measured using the Walk-Way MW-1000, as it has been reported to influence gait variability^8,9)^.

## Follow-up and Outcomes

Table 1 summarizes the progress across evaluations. Physical therapy and gait variability measurements were conducted at 1, 2, 3, 4, and 5 months post-stroke during hospitalization, with follow-up assessments at 8 and 24 months. Upon discharge (5 months post-stroke), the patient’s weight had decreased to 96.5 kg, resulting in a BMI of 33.4 kg/m^2^. The patient had no recurrent strokes for 24 months after the onset of the stroke. At discharge, the FMA scores indicated improvement to moderate motor paresis, with scores of 30 for both the upper and lower extremities^17)^. This patient continued experiencing a slightly reduced sensation on the right side. The patient’s FAC improved to 4 at 4 months and to 5 at 5 months. The 6MWT improved to 390 m without using a cane. Additionally, the BBS score increased to 53 points, exceeding the 50.5-point cutoff for fall risk^23)^. The ABC-J demonstrated an average score of 69.4%, indicating an improved balance efficacy. After achieving independent walking at 4 months post-stroke, the patient’s PA increased to 6231 steps/day. During discharge, his daily step count had increased to 6530 steps/day, exceeding the 6025 steps recommended for preventing vascular event recurrence^24)^. No adverse events occurred during the rehabilitation process. The patient returned to work following discharge. At follow-up 24 months post-stroke, the patient noted improvements in physical function, PA, and balance efficacy. Notably, the patient successfully walked independently in outdoor settings, requiring no assistive devices, including a cane or an AFO, for support.

**Table 1.** Progression of physical function, walking ability, balance, self-efficacy, and physical activities during the 24 months post-stroke

	1 month	2 months	3 months	4 months	5 months	8 months	24 months
FMA-LE	13	19	23	26	30	31	32
FAC	2	2	3	4	5	5	5
6MWT (m)	200	250	287	356	390	432	NR
BBS	33	42	46	50	53	56	56
ABC-J (%)	8.8	18.1	30.0	60.0	69.4	76.9	88.1
PA (steps/day)	54	27	26	6231	6530	7099	7562

FMA-LE, Fugl-Meyer Assessment for the lower extremities; FAC, functional ambulation categories; 6MWT, 6-minute walk test; m, meter; BBS, Berg Balance Scale; ABC-J, Activities-specific Balance Confidence scale-Japanese; PA, physical activities; NR, not reported

### Gait variability and speed progression

Table 2 presents the temporal progression of the GVI, the CV for each spatiotemporal gait parameter, and the walking speed. First, at 1 month post-stroke, the GVI scores were 62.1 and 66.9 on the paretic and nonparetic sides, respectively, with an average score of 64.5. At 4 months post-stroke, the GVI scores peaked at 88.0 and 95.8 on the paretic and nonparetic sides, respectively, with an average score of 91.9. Follow-up assessments following discharge revealed no further notable improvements. Second, at 1 month post-stroke, the CV of each spatiotemporal gait parameter showed higher variability. A subsequent reduction in CV was observed, with no changes noted on either side after 4 months post-stroke (Supplementary Material 1). Analysis of the rate of decrease in the average CV of the paretic and nonparetic sides from 1 to 24 months post-stroke revealed that the step length and single support time (swing time) demonstrated the greatest reductions (−77.0% and −73.5%, respectively). Lastly, an improvement in walking speed was observed, increasing from 0.36 m/s at 1 month post-stroke to 1.11 m/s at 24 months post-stroke.

**Table 2.** Progression of gait variability and gait speed during the 24 months post-stroke

	1 month	2 months	3 months	4 months	5 months	8 months	24 months
GVI							
Average	64.5	66.8	75.0	91.9	90.7	90.9	90.9
Paretic side	62.1	65.1	74.8	88.0	88.9	88.9	88.1
Nonparetic side	66.9	68.4	75.2	95.8	92.4	92.9	93.7
Stride velocity CV (%)	14.4	13.9	11.9	3.39	1.84	4.27	4.58
Stride time CV (%)	5.92	7.27	7.52	2.85	1.65	2.04	2.29
Step time CV (%)	6.77	6.92	7.12	2.76	3.15	2.35	3.08
Swing time, single support time CV (%)	14.2	10.1	7.68	4.11	4.35	4.23	3.76
Stance time CV (%)	7.94	8.62	7.90	2.11	3.58	3.00	2.89
Double support time CV (%)	15.5	10.4	11.5	5.29	5.69	5.27	6.13
Stride length CV (%)	12.2	10.2	8.32	2.12	3.11	3.88	3.29
Step length CV (%)	21.0	14.5	9.88	2.36	3.80	6.05	4.83
Walking speed (m/s)	0.36	0.47	0.54	0.85	0.85	0.98	1.11

CV data are presented as averages for the paretic and nonparetic sides.

GVI, Gait Variability Index; CV, coefficient of variation; m, meter; s, second

## Discussion

This case report described the improvement in gait variability in an individual with hemiparesis following stroke over 24 months. The findings indicate that gait variability achieved its greatest improvement during the subacute phase, 4 months post-stroke, with no substantial changes observed thereafter. Among the variability in spatiotemporal gait parameters, step length, single support time, and swing time demonstrated the most marked reductions. These findings provide knowledge about the process of improvement of gait variability in people with stroke and offer potential developments for future studies.

The longitudinal tracking of gait variability in an individual with hemiparesis following stroke represents the novel aspect of this report. Previous studies have predominantly focused on immediate or short-term changes^6)^. Another study measured gait variability over 48 weeks post-stroke, but participants were limited to those who could sit-to-stand independently^25)^. In contrast, this report provides a longitudinal perspective on gait variability, monitoring changes from 1 to 24 months post-stroke, when fall risk is present and assisted walking is required. Although the findings are based on a single case, they may aid clinicians and patients in understanding the trajectory of gait variability in patients with hemiparesis following stroke over extended periods.

The improvement in gait variability in this patient does not necessarily coincide with an increase in speed. It was initially hypothesized that gait variability could continue to improve beyond the subacute phase. This hypothesis was based on previous findings suggesting that walking speed may improve even after the subacute phase in patients with stroke^26)^. The variability of many spatiotemporal parameters increases as walking speed decreases, and walking speed correlates with the variability of step time, step length, and step width^9,27)^. However, previous studies have indicated that gait speed and gait variability, measures of gait function and control, respectively, are not necessarily directly associated^22)^. Furthermore, recent studies suggest that variability in single support time and step velocity may not be directly influenced by walking speed^9,27)^. In this patient, although gait variability reached its maximum improvement at 4 months post-stroke, walking speed showed further increase over the 24-month period. These findings suggest that although increased walking speed may contribute to improvements in gait variability, the two follow different long-term patterns, with variability improving more quickly than speed. Furthermore, because the recovery progress in gait variability and speed may differ^25)^, it may be useful in clinical practice to assess walking ability using gait variability indicators in addition to walking speed.

The observed improvements in single support time variability and step length variability may reflect the recovery from the motor deficits caused by the stroke. Single support time variability is specifically increased following stroke onset^9)^, and increased step length variability has been reported to indicate foot placement errors during walking owing to motor deficits^11)^. In patients with stroke, the severity of motor deficits has been shown to influence gait variability, with moderate and severe cases exhibiting increased variability compared with mild cases^7)^. In the present case, the FMA lower extremity score substantially increased, from 13 points at 1 month post-stroke to 30 and 32 points at 5 and 24 months following stroke, respectively. Previous studies have reported that the minimal detectable change in FMA lower extremity scores for patients with stroke ranges from 1.24 to 3.23 points^28)^, with the minimal clinically important difference set at 6 points^29)^. In this patient, recovery exceeded these thresholds, suggesting that improvements in motor deficits contributed to the observed reductions in gait variability.

The improvement in gait variability observed in the patient may be associated with the attainment of independent walking, elevated PA levels, and gait training. Previous studies have reported that the stride duration CV during walking is correlated with PA levels in patients with stroke^30)^. This patient achieved independent walking at 4 months post-stroke, consistent with the maximum improvement in gait variability and increased PA levels. These findings suggest that improvements in gait variability may provide a foundation for achieving independent walking and promoting PA. Moreover, appropriate BWS during walking reduces variability and enhances dynamic stability^31)^. However, as this report describes a single clinical case, causality between gait variability, independent walking, PA, and gait training cannot be established.

This case report has several limitations. As this is a single case report, the results cannot be generalized. Furthermore, the patient did not undergo regular checkups after discharge, and his gait was measured only when he visited the hospital. Thus, there was a lack of regular, continuous data over 24 months. Therefore, future studies should aim to increase the number of cases to ensure the reliability of measurements and examine the longitudinal progress of gait variability.

## Conclusions

This case report described the improvement in gait variability over 24 months in an individual with hemiparesis following stroke. At 4 months post-stroke, this patient’s gait variability achieved its maximum improvement, with step length and single support time variability exhibiting the most improvements.

## Supplementary Material

Supplementary Material 1. Changes in the coefficient of variation in spatiotemporal gait parameters of the paretic and nonparetic limbs over 24 months post-stroke.
