# Supplementary material for: Improvement in Gait Variability over 24 Months in an Individual with Hemiparesis following Stroke: A Case Report
Source: Phys Ther Res. 2026 Feb 6;29(1):60–5. doi: 10.1298/ptr.25-E10380 (PMC13143133; doi:10.1298/ptr.25-E10380)
Supplement: Supplementary file 1 — Supplementary Material 1. Changes in the coefficient of variation in spatiotemporal gait parameters of the paretic and nonparetic limbs over 24 months post-stroke. [file ptr-29-60-s001.pdf]

Supplementary Material 1. Changes in the coefficient of variation in spatiotemporal gait parameters of the paretic and nonparetic limbs over 24 months post-stroke.

	1 month	2 months	3 months	4 months	5 months	8 months	24 months
Stride Velocity CV (%)							
Paretic side	6.67	12.7	12.0	4.17	2.15	3.72	4.72
Non-paretic side	22.1	15.1	11.8	2.62	1.54	4.83	4.45
Stride time CV (%)							
Paretic side	5.17	9.24	8.00	3.11	1.89	1.73	2.08
Non-paretic side	6.67	5.30	7.04	2.59	1.41	2.36	2.50
Step time CV (%)							
Paretic side	7.10	6.91	9.18	3.07	1.45	2.70	3.40
Non-paretic side	6.44	6.93	5.07	2.45	4.85	2.00	2.76
Swing time, Single support time CV (%)							
Paretic side	17.3	12.0	8.32	6.23	3.89	4.40	4.83
Non-paretic side	11.1	8.27	7.04	1.99	4.80	4.06	2.69
Stance time CV (%)							
Paretic side	7.37	10.8	7.77	1.71	3.58	2.62	2.59
Non-paretic side	8.51	6.42	8.04	2.50	3.59	3.38	3.19
Double support time CV (%)							
Paretic side	19.5	9.49	13.0	4.36	5.31	4.08	4.83
Non-paretic side	11.4	11.4	9.92	6.21	6.06	6.47	7.42
Stride length CV (%)							
Paretic side	6.66	8.03	7.24	2.44	3.64	3.37	3.24
Non-paretic side	17.8	12.5	9.41	1.80	2.58	4.39	3.33
Step length CV (%)							
Paretic side	21.8	10.5	10.5	1.69	2.44	6.33	4.80
Non-paretic side	20.3	18.4	9.23	3.02	5.16	5.77	4.85

CV, Coefficient of variation.
